# Thromboembolic Risk of 4-Factor Prothrombin Complex Concentrate versus Fresh Frozen Plasma for Urgent Warfarin Reversal in the Emergency Department

**DOI:** 10.5811/westjem.2019.4.41649

**Published:** 2019-06-11

**Authors:** Michelle Maguire, Lanting Fuh, Joshua N. Goldstein, Ariela L. Marshall, Michael Levine, Melissa L. Howell, Blair A. Parry, Rachel Rosovsky, Bryan D. Hayes

**Affiliations:** * Massachusetts General Hospital, Department of Pharmacy, Boston, Massachusetts; † Massachusetts General Hospital, Department of Emergency Medicine, Boston, Massachusetts; ‡ Massachusetts General Hospital, Center for Vascular Emergencies, Boston, Massachusetts; § Massachusetts General Hospital, Division of Hematology, Department of Internal Medicine, Boston, Massachusetts; ¶ Harvard Medical School, Department of Emergency Medicine, Boston, Massachusetts; || Harvard Medical School, Department of Internal Medicine, Boston, Massachusetts; # Mayo Clinic, Division of Hematology, Department of Internal Medicine, Rochester, Minnesota; ** Mayo Clinic, Department of Laboratory Medicine and Pathology, Rochester, Minnesota; †† University of Southern California, Department of Emergency Medicine, Department of Medical Toxicology, Los Angeles, California; ‡‡ University of the South, The School of Theology, Sewanee, Tennessee

## Abstract

**Introduction:**

Warfarin is a potent anticoagulant used for the prevention and treatment of venous and arterial thrombosis. Occasionally, patients require emergent warfarin reversal due to active bleeding, supratherapeutic international normalized ratio, or emergent diagnostic or therapeutic interventions. Various agents can be used for emergent warfarin reversal, including fresh frozen plasma (FFP) and 4-factor prothrombin complex concentrate (4F-PCC). Both FFP and 4F-PCC are generally considered safe; however, both agents contain coagulation factors and have the potential to provoke a thromboembolic event. Although clinical trials have compared the efficacy and safety of FFP and 4F-PCC, data are limited comparing the risk of thromboembolism between the two agents.

**Methods:**

A retrospective chart review was performed at a single, urban, academic medical center comparing the incidence of thromboembolism with FFP or 4F-PCC for warfarin reversal during a three-year period in the emergency department (ED) at Massachusetts General Hospital. Patients were included in the study if they were at least 18 years of age and were on warfarin per electronic health records. Patients were excluded if they had received both FFP and 4F-PCC during the same visit. The primary outcome was the frequency of thromboembolism within 30 days of 4F-PCC or FFP. Secondary outcomes included time to thromboembolic event and in-hospital mortality.

**Results:**

Three hundred and thirty-six patients met the inclusion criteria. Thromboembolic events within 30 days of therapy occurred in seven patients (2.7%) in the FFP group and 14 patients (17.7%) in the 4F-PCC group (p=<0.001). Death occurred in 39 patients (15.2%) who received FFP and 18 patients (22.8%) who received 4F-PCC (p=0.115). Since the 4F-PCC group was treated disproportionately for central nervous system (CNS) bleeding, a subgroup analysis was performed including patients requiring reversal due to CNS bleeds that received vitamin K. The primary outcome remained statistically significant, occurring in four patients (4.1%) in the FFP group and nine patients (14.1%) in the 4F-PCC group (p=0.02).

**Conclusion:**

Our study found a significantly higher risk of thromboembolic events in patients receiving 4F-PCC compared to FFP for urgent warfarin reversal. This difference remained statistically significant when controlled for CNS bleeds and administration of vitamin K.

## INTRODUCTION

Warfarin is a potent anticoagulant used for the prevention and treatment of venous and arterial thrombosis. As a vitamin K antagonist, warfarin prevents the post-translational carboxylation of coagulation factors II, VII, IX, and X, along with protein C and S, by 30–50%.^1^ Warfarin is one of the top medications implicated in emergency department (ED) visits due to bleeding events. Annually, bleeding complications associated with over-anticoagulation with warfarin occur in 15–20% of patients, with fatal bleeds accounting for 1–3%.^2^

Occasionally, patients require emergent warfarin reversal due to life-threatening bleeds or the need for emergency diagnostic or therapeutic intervention. The risk of bleeding is directly related to the degree of international normalized ratio (INR) elevation.^3^ It is important to note, however, that half of all major bleeding episodes associated with warfarin occur when the INR is less than 4.0.^4^ The degree and rapidity of reversal are dependent upon not only the absolute value of the INR, but also the clinical indication for reversal. Prior to the approval of 4-factor-prothrombin complex concentrate (4F-PCC) in 2013, fresh frozen plasma (FFP) was the preferred therapy for reversing warfarin in the United States. However, 4F-PCC reduces the INR more quickly and is now preferred as a first-line agent for warfarin reversal in intracranial hemorrhage, the most disabling form of major bleeding.^5^,^6^,^7^,^8^,^9^ Both FFP and 4F-PCC are generally considered safe; however, both agents contain coagulation factors and have the potential to provoke a thromboembolic event. Although clinical trials have compared the efficacy and safety of FFP and 4F-PCC, there is sparse data comparing the risk of thromboembolism between the two agents outside the clinical trial setting, using “real world” data. 6,7,10,11,12,13,14

The primary objective of this study was to assess the incidence of thromboembolic events in patients who received either 4F-PCC or FFP for emergent warfarin reversal.

## METHODS

We performed a retrospective chart review at a single, urban, academic medical center. Consecutive patients receiving 4F-PCC in the ED between April 20, 2016 – October 28, 2017, or FFP in the ED between January 1, 2010 – January 30, 2011, were identified from the electronic health record (EHR). During the FFP period, 4F-PCC was not available for use at our institution, and FFP was used for all emergent warfarin reversal. In April 2013, 4F-PCC became available for warfarin reversal, although its use was restricted to patients with central nervous system (CNS) and intrapulmonary bleeds on warfarin. Any other indications required hematology approval. A report from the hospital’s EHR identified patients who received an order for FFP or 4F-PCC while in the ED.

Patients were included in the current study if they were at least 18 years of age and were on warfarin as per EHR, including outpatient medication lists, previous prescriptions, and prescriber notes. We excluded patients if they had received both FFP and 4F-PCC during the same visit. Collected data included initial INR on presentation to the ED, indication for warfarin, indication for warfarin reversal, administration and dose of FFP or 4F-PCC, administration of vitamin K, thromboembolic events, and mortality. Age, gender, and race were also collected for demographic purposes. Thromboembolic events were identified by reviewing provider notes, discharge summaries, follow-up notes, imaging, and medication administration records.

**Population Health Research Capsule**

What do we already know about this issue?

Despite clinical trials of fresh frozen plasma (FFP) vs. 4-factor prothrombin complex concentrate (4F-PCC), few data compare thromboembolism risk for warfarin reversal.

What was the research question?

What is the incidence of thromboembolism using FFP compared to 4F-PCC for urgent warfarin reversal?

What was the major finding of the study?

There was a higher risk of thromboembolic events in patients receiving 4F-PCC compared to FFP for urgent warfarin reversal.

How does this improve population health?

While 4F-PCC is the preferred agent for warfarin reversal, providers must weigh the risks and benefits when using it in patients already at high risk of thromboembolism.

We used the search function of the EHR to ensure that no thromboembolic events occurring at our hospital were overlooked. This search function identifies the searched term in the EHR, as well as any other medically related terms. For example, when searching for the term “clot,” similar search terms such as thromboembolism and thrombus are also identified. Patients in the FFP group were originally collected for a separate analysis of pulmonary complication rates after FFP administration and time to INR reversal.^15^

Abstractors were trained in clinical research and quality assurance. There was an initial review of study variables, standard operating procedures for data abstraction, and the data abstraction form (which was electronic, using REDCap). Each abstractor then reviewed a set of practice records, with variables verified for accuracy and discrepancies adjudicated. After training, regular meetings occurred to review data collection and address questions and discrepancies.

The primary outcome was the frequency of thromboembolism within 30 days of 4F-PCC or FFP administration. Secondary outcomes included time to thromboembolic event and in-hospital mortality.

### Statistical Analysis

This study was approved by the institutional review board. We collected and analyzed data using RedCap and Excel. We used chi-square tests to compare the rate of thromboembolic events and mortality between patients who received 4F-PCC and FFP. The method by Cohen was used to determine power since there were no previous studies on which to base our power analysis.^16^ In order to detect a medium effect size difference (d = 0.5 where d = (mean_a_-mean_b_)/α) and α = 0.05), it was estimated that 64 patients would be required for each group. We also calculated Cohen’s kappa to assess for inter-rater reliability. This number was based on 10% of our data chosen at random to be reabstracted by an independent reviewer for our primary endpoint.

## RESULTS

**Figure 1:**
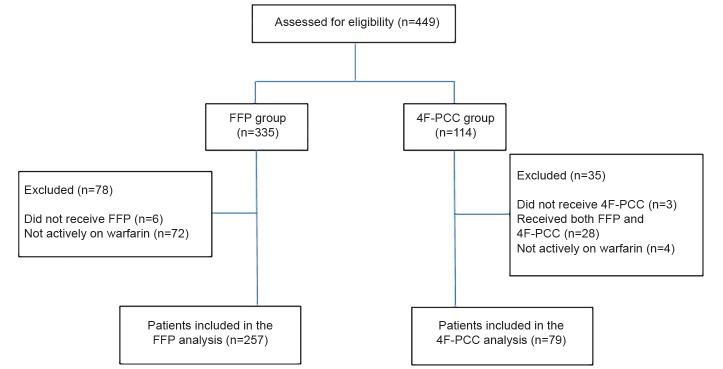
Study inclusion and exclusion. *FFP,* fresh frozen plasma; *4F-PCC*, 4-factor prothrombin complex concentrate.

Table 1 shows patients’ baseline characteristics. The most common reason for patients to receive 4F-PCC was CNS bleed (82.3%), while the indications for FFP were more varied, including CNS bleed (38.1%), urgent surgery (25.7%), and gastrointestinal bleed (19.8%). The median amount of FFP administered was 3 units (interquartile range [IQR] 1–3) with the average dose being 9.4 milliliters per kilogram (mL/kg). The mean dose of 4F-PCC administered was 2168 units (standard deviation [SD] 723 units). All patients included in the 4F-PCC group received a dose within 10% of their calculated dose based on their initial INR and weight, consistent with U.S. Food and Drug Administration (FDA)-approved dosing. Inclusion and exclusion criteria are described in Figure 1.

The primary outcome, thromboembolic events within 30 days, occurred in seven patients (2.7%) in the FFP group and 14 patients (17.7%) in the 4F-PCC group (p=<0.001). We calculated a Cohen’s kappa score of 0.84 based on 10% of the data reabstracted by an independent reviewer. The mean time to thromboembolic event was 4.1 days (SD 5.4) in the FFP group and 8.9 days (SD 8.7) in the 4F-PCC group (p=0.20). We also evaluated thromboembolic events based on type and indications for warfarin use, as seen in Tables 2 and 3. Two patients in the FFP group and four patients in the 4F-PCC group had superficial clots, all of which were cephalic vein thromboses. When these less dangerous and non-life-threatening clots were removed from the analysis, the difference in thromboembolic events remained statistically significant (p=<0.001) between the two groups.

Vitamin K was administered in 209 of 257 (81.3%) patients in the FFP group and 78 of 79 (98.7%) patients in the 4F-PCC group (p=0.0002). Death occurred in 39 patients (15.2%) who received FFP and 18 patients (22.8%) who received 4F-PCC (p=0.115). Cause of death in the FFP group was attributed to a bleeding event in 20 patients and thromboembolic event in two patients, while 17 patients had other, non-related or unclear causes of death. In the 4F-PCC group, death was attributed to a bleeding event in 10 patients and thromboembolic event in two patients, while six patients had other, non-related or unclear causes of death. All bleeding events resulting in death were attributed to the presenting event.

As the 4F-PCC group was treated disproportionately for CNS bleeding (due to the hospital guideline restricting 4F-PCC use to high-risk conditions such as this), we performed a subgroup analysis including only patients requiring warfarin reversal due to CNS bleeds and those that received vitamin K in the ED. There were 98 patients included in the FFP group and 65 patients included in the 4F-PCC group. The median amount of FFP administered was 4 units (IQR 1–3), while the mean dose of 4F-PCC administered was 2148 units (SD 698 units). The primary outcome, thromboembolic events within 30 days, remained statistically significant occurring in four patients (4.1%) in the FFP group and nine patients (14.1%) in the 4F-PCC group (p=0.02). Death occurred in 30 patients (30.6%) who received FFP and 17 patients (26.2%) who received 4F-PCC (p=0.54).

## DISCUSSION

According to the American College of Cardiology (ACC) and the Neurocritical Care Society guidelines on anticoagulation reversal, 4F-PCC is currently recommended as the preferred method for emergency warfarin reversal.^8^,^9^ Although patients receiving warfarin have pre-existing thromboembolic risk factors that may be unmasked with reversal, it is not clear whether different warfarin reversal options carry different thromboembolic risks. Phase 2 and 3 clinical trials evaluating thromboembolic events between FFP and 4F-PCC for vitamin K antagonist reversal found no difference between these options.^5^,^6^,^7^,^17^ However, our study of real-world data suggests the thromboembolic risk of 4F-PCC may be higher than FFP. Importantly, the thromboembolic events in the 4F-PCC group, on average, occurred much later in the clinical course and therefore may be less related to the initial reversal administered. Although this difference was not statistically significant, it could have clinically significant implications.

Several factors may have contributed to the difference in thromboembolic risk in our study, the first of which is the differences in baseline characteristics. Patients who received 4F-PCC had a significantly higher rate of atrial fibrillation requiring warfarin therapy, while more FFP patients were under treatment for thromboembolic disorders such as pulmonary emboli. Patients in the FFP group had an overall higher risk of venous thromboemboli, potentially putting them at higher risk of thromboembolic events with warfarin reversal. Our results demonstrated the opposite, suggesting that the patients in the 4F-PCC group may have an increased risk of thromboembolic events despite their indication for warfarin.

The dose of FFP administered and differences in vitamin K administration may also have contributed. When using FFP for the emergency reversal of warfarin, 10–15mL/kg is recommended.^9^ Most adults will need an average dose of 3–6 units to replace enough coagulation factors to reverse warfarin. In our study, the median amount of FFP given was 3 units, with the group having a mean weight of 79.8 kg. On average, patients in our study received a subtherapeutic dose of FFP at 9.4 mL/kg, while all patients in the 4F-PCC group received the recommended dose based on their weight and INR. For comparison, the FFP arms of clinical trials include doses that are substantially higher than those used in our center during the study period;^6^,^7^,^17^ therefore, our FFP patients may have been exposed to lower thromboembolic risk. Although the average FFP dose administered in our study was subtherapeutic according to the guidelines, it was within 10% of the lower limit of the recommended dose of 10 mL/kg, which suggests that the lower end of the dosing range may be safer.

The difference in Vitamin K administration might also have contributed to the difference in thromboembolic events. The FFP group received vitamin K significantly less often than the 4F-PCC group (81% vs.99%, respectively; p = <0.001), a difference that was not seen in most other studies.^5^,^6^,^7^

Another difference identified between the two groups was the number of CNS bleeds. Our hospital guideline allows 4F-PCC to be ordered without specialist approval for patients with CNS bleeds; however, most other uses require hematology approval. To control for this, we performed a subgroup analysis examining only those with CNS bleeds who received vitamin K. The thromboembolic event rate remained higher in the 4F-PCC group. However, there was no difference in mortality. It remains possible that providers selected more severely injured patients for 4F-PCC.^18^

Several studies examined the difference between thromboembolic events as a secondary outcome, but only a few have looked at this occurrence as a primary outcome.^5^,^6^,^7^,^17^ A post hoc exploratory analysis of two randomized controlled trials found that most thromboembolic events in the 4F-PCC group occurred >7 days after reversal, clustering around the two-week mark while thromboembolic events in the FFP group occurred within seven days of reversal, with nearly 50% occurring within the same day. This difference may be due to the increased amount of vitamin K and non-vitamin K dependent coagulation factors being loaded over a short period but also raises the question of whether the thromboembolic events were caused by the administration of 4F-PCC or a consequence of prolonged hospitalizations and delayed anticoagulation initiation after a major bleeding event.

The ACC published an expert consensus on the management of bleeding in patients on oral anticoagulants, in which they recommend the use of either variable dosing of 4F-PCC based on INR and weight or fixed dose.^9^ There are few data to suggest that the rate of thromboembolic events with 4F-PCC is dose dependent. However, giving a fixed dose of 1000–1500 international units may theoretically reduce the risk of thromboembolic events. Since our study used the variable FDA dosing of 4F-PCC based on INR and weight, further studies are needed to determine if the risk of thromboembolic events would remain significant between FFP and 4F-PCC when using fixed dose 4F-PCC.

## LIMITATIONS

Limitations of this study include its retrospective, single-center design, which only included patients seen and followed up at our hospital. Although we were able to identify all patients that followed up at hospitals within our healthcare system, patients may have been missed if they had thromboembolic events or died at hospitals outside of our system. Another limitation is the difference in time periods in which the data were collected and the difference in baseline characteristics between the two groups. Although 4F-PCC was FDA approved in 2013, its distribution was managed by the blood bank at our institution until 2016, when it was transferred to the pharmacy department.^18^ Due to this timing, we were unable to obtain any data for 4F-PCC use before this time. To ensure inter-rater reliability an independent abstractor conducted quality assurance using the Cohen’s kappa score by reabstracting 10% of the data. Despite the difference in abstractors and time periods, a kappa score of 0.84 suggested almost perfect agreement between the abstractors. In addition, clinical care of patients requiring warfarin reversal may have changed during the study period, and data capture for thromboembolic events may have improved, artificially increasing the frequency of thromboembolism over time. Lastly, we are unable to comment on the long-term morbidity and mortality between the two groups as this study only analyzed data up to 30 days after patients received 4F-PCC and FFP.

## CONCLUSION

Our study found a higher risk of thromboembolic events in patients receiving FDA-approved doses of 4F-PCC compared to FFP for urgent warfarin reversal. This difference remained when controlled for CNS bleeds and administration of vitamin K. Thromboembolic events, on average, developed several days later in the 4F-PCC group compared to the FFP group. Although 4F-PCC is the preferred agent for emergent warfarin reversal, it is important for providers to weigh the risks and benefits when using this agent in patients already at high risk of thromboembolic events.

## Figures and Tables

**Table 1:** Baseline characteristics.

	4F-PCC (n=79)	FFP (n=257)	p-value
Sex			
Male	59.5%	59.1%	0.96
Female	40.5%	40.9%	0.96
Age (years)	75.2	73.0	0.09
Race			
Hispanic/Latino	2 (2.5%)	4 (1.6%)	0.57
Not Hispanic/Latino	76 (96.2%)	252 (98.0%)	0.35
Unavailable	1 (1.3%)	1 (0.4%)	0.38
Weight (kg)	78.3	79.8	0.26
Baseline INR	3.65	3.86	0.37
Indication for Warfarin*			
Atrial fibrillation	60 (75.9%)	162 (63.0%)	0.03
Mitral valve replacement	4 (5.1%)	7 (2.7%)	0.31
Aortic valve replacement	6 (7.6%)	13 (5.1%)	0.39
Deep vein thrombosis	7 (8.8%)	41 (15.9%)	0.12
Pulmonary embolism	4 (5.1%)	36 (14.0%)	0.03
Hypercoagulable state	5 (6.3%)	11 (4.3%)	0.45
Other	3 (3.8%)	57 (22.1%)	<0.001
Indication for Warfarin reversal*			
CNS bleed	65 (82.3%)	98 (38.1%)	<0.001
GI bleed	5 (6.3%)	51 (19.8%)	0.005
Musculoskeletal bleed	3 (3.8%)	2 (0.8%)	0.05
Intra-abdominal bleed	0 (0%)	10 (3.9%)	0.13
Hematuria	0 (0%)	4 (1.6%)	0.58
Hemoptysis	0 (0%)	3 (1.2%)	1.0
Epistaxis	0 (0%)	2 (0.8%)	1.0
Hematoma	4 (5.1%)	4 (1.6%)	0.07
Hemothorax	0 (0%)	1 (0.4%)	1.0
Other bleeding**	0 (0%)	7 (2.7%)	0.21
Surgery	4 (5.1%)	66 (25.7%)	<0.001
Other indications for reversal	0 (0%)	7 (2.7%)	0.21
Average dose	28 IU/kg	9.4 mL/kg	
Concomitant vitamin K	78 (98.7%)	209 (81.3%)	<0.001

* Numbers do not add up to 100% as some patients had more than one indication for warfarin or warfarin reversal.

** Other bleeding includes vaginal bleeding, catheter site bleeding, arteriovenous fistula, hemorrhagic ovarian cyst, hemorrhagic goiter, bleeding associated with multi-trauma, and hemathrosis.

*FFP*, fresh frozen plasma; *4F-PCC*, 4-factor prothrombin complex concentrate; *kg*, kilogram;* INR*, international normalized ratio; *CNS*, central nervous system; *GI*, gastrointestinal; *mL/kg*, milliliters per kilogram; *IU/kg*, international unit per kilogram.

**Table 2:** Thromboembolic events within 30 days of warfarin reversal.

	4F-PCC (14)	FFP (7)	p-value
Myocardial infarction	0 (0%)	1 (14.3%)	0.33
Cerebral vascular accident	2 (14.3%)	0 (0%)	0.53
Pulmonary embolism	2 (14.3%)	1 (14.3%)	1
Deep venous thrombosis	4 (28.6%)	3 (42.9%)	0.64
Superficial thrombosis*	4 (28.6%)	2 (28.6%)	1
Other** thromboembolic event	2 (14.3%)	0 (0%)	0.53

* Superficial thrombosis includes cephalic vein thrombus.

** Other events include left ventricular thrombus and right atrial thrombus.

*FFP,* fresh frozen plasma; *4F-PCC*, 4-factor prothrombin complex concentrate.

**Table 3:** Warfarin indications for patients with thromboembolic events after reversal with 4-factor prothrombin complex concentrate (4F-PCC) or fresh frozen plasma (FFP).

Thromboembolic event	Warfarin Indication*	

	4F-PCC	FFP
	
Cerebral vascular accident (n=1 in 4F-PCC)	Atrial fibrillation (n=1) Cerebral vascular accident (n=1)	None
Myocardial infarction (n=1)	None	Atrial fibrillation (n=1)
Pulmonary embolism (n=2 in 4F-PCC and 1 in FFP)	Atrial fibrillation (n=2) Deep venous thrombosis (n=1)	Deep venous thrombosis (n=1) Hypercoagulable state (n=1)
Deep venous thrombosis (n=4 in 4F-PCC and 4 in FFP)	Atrial fibrillation (n=4) Deep venous thrombosis (n=1) Factor V Leiden (n=1)	Atrial Fibrillation (n=3) Deep venous thrombosis (n=1) Hypercoagulable state (n=2)
Superficial thrombosis (n= 4 in 4F-PCC and 2 in FFP)	Mechanical valve (n=2) Atrial fibrillation (n=3)	Pulmonary embolism (n=1) Deep venous thrombosis (n=1) Hypercoagulable state (n=1)
Other thromboembolic event (n=2 in 4F-PCC)	Atrial fibrillation (n=1) Mechanical valve (n=1) Deep venous thrombosis (n=1)	None

* Warfarin indications were not mutually exclusive.

*FFP,* fresh frozen plasma; *4F-PCC*, 4-factor prothrombin complex concentrate.
